# Modifiable causes of premature death in middle-age in Western Europe: results from the EPIC cohort study

**DOI:** 10.1186/s12916-016-0630-6

**Published:** 2016-06-14

**Authors:** David C. Muller, Neil Murphy, Mattias Johansson, Pietro Ferrari, Konstantinos K. Tsilidis, Marie-Christine Boutron-Ruault, Francoise Clavel, Laureen Dartois, Kuanrong Li, Rudolf Kaaks, Cornelia Weikert, Manuela Bergmann, Heiner Boeing, Anne Tjønneland, Kim Overvad, M. Luisa Redondo, Antonio Agudo, Elena Molina-Portillo, Jone M. Altzibar, Lluís Cirera, Eva Ardanaz, Kay-Tee Khaw, Nicholas J. Wareham, Timothy J. Key, Ruth C. Travis, Christina Bamia, Philippos Orfanos, Antonia Trichopoulou, Domenico Palli, Valeria Pala, Rosario Tumino, Paolo Vineis, Salvatore Panico, H. Bas Bueno-de-Mesquita, W. M. Monique Verschuren, Ellen A. Struijk, Petra H. Peeters, Gunnar Engström, Olle Melander, Malin Sund, Elisabete Weiderpass, Guri Skeie, Eiliv Lund, Teresa Norat, Marc Gunter, Elio Riboli, Paul Brennan

**Affiliations:** International Agency for Research on Cancer, 69008 Lyon, France; School of Public Health, Imperial College London, London, SW7 2AZ UK; Department of Hygiene and Epidemiology, University of Ioannina School of Medicine, 45110 Ioannina, Greece; Department of Epidemiology and Biostatistics, School of Public Health, Imperial College London, London, SW7 2AZ UK; Centre for Research in Epidemiology and Population Health (CESP), 94805 Villejuif, France; Université Paris Sud, Villejuif, France; Institut Gustave Roussy, Villejuif, France; Inserm, Centre for Research in Epidemiology and Population Health (CESP), 94805 Villejuif, France; Division of Cancer Epidemiology, German Cancer Research Center (DKFZ), DE-69120 Heidelberg, Germany; German Institute of Human Nutrition Potsdam-Rehbrücke (DifE), DE-14558 Nuthetal, Germany; Danish Cancer Society Research Center, DK-2100 Copenhagen, Denmark; Department of Public Health, Section for Epidemiology, Aarhus University, DK-8000 Aarhus, Denmark; Public Health Directorate, 33006 Asturias, Spain; Unit of Nutrition, Environment and Cancer, Cancer Epidemiology Research Program, Catalan Institute of Oncology, 08908 Barcelona, Spain; Escuela Andaluza de Salud Pública, Instituto de Investigación Biosanitaria GRANADA, Hospitales Universitarios de Granada/Universidad de Granada, 18012 Granada, Spain; Consortium for Biomedical Research in Epidemiology and Public Health (CIBER Epidemiología y Salud Pública-CIBERESP), Madrid, Spain; Public Health Division of Gipuzkoa-BIODONOSTIA, Basque Regional Health Department, 20014 Donostia - San Sebastián, Spain; Department of Epidemiology, Murcia Regional Health Council, IMIB-Arrixaca, 30003 Murcia, Spain; Department of Health and Social Sciences, Universidad de Murcia, Murcia, Spain; Navarra Public Health Institute, 31003 Pamplona, Spain; Navarra Institute for Health Research (IdiSNA) Pamplona, Pamplona, Spain; School of Clinical Medicine, University of Cambridge, Cambridge, CB2 2QQ UK; MRC Epidemiology Unit, University of Cambridge, Cambridge, CB2 0SR UK; Cancer Epidemiology Unit, University of Oxford, Oxford, OX3 7LF UK; Hellenic Health Foundation, GR-115 27 Athens, Greece; Department of Hygiene, Epidemiology and Medical Statistics, University of Athens Medical School, Athens, Greece; Molecular and Nutritional Epidemiology Unit, Cancer Research and Prevention Institute (ISPO), 50134 Florence, Italy; Epidemiology and Prevention Unit, Fondazione IRCCS Istituto Nazionale dei Tumori, 20133 Milan, Italy; Cancer Registry and Histopathology Unit, “Civic - M. P. Arezzo” Hospital, ASP Ragusa, Ragusa, 97100 Italy; Human Genetics Foundation (HuGeF), Torino, Italy; Dipartimento di Medicina Clinica e Sperimentale, Federico II University, 80138 Naples, Italy; Department for Determinants of Chronic Diseases (DCD), National Institute for Public Health and the Environment (RIVM), 3720 BA Bilthoven, The Netherlands; Department of Gastroenterology and Hepatology, University Medical Centre, Utrecht, The Netherlands; Department of Social and Preventive Medicine, Faculty of Medicine, University of Malaya, Kuala Lumpur, Malaysia; Department of Epidemiology, Julius Center for Health Sciences and Primary Care, University Medical Center Utrecht, Utrecht, The Netherlands; Department of Clinical Science, Malmö Lund University, Lund, SE-205 02 Sweden; Department of Clinical Sciences, Hypertension & Cardiovascular Disease, Clinical Research Centre, Malmö University Hospital, SE-20502 Malmö, Sweden; Department of Surgical and Perioperative Sciences, Umea University, 901 85 Umea, Sweden; Department of Community Medicine, Faculty of Health Sciences, University of Tromsø, The Arctic University of Norway, 9037 Tromsø, Norway; Department of Research, Cancer Registry of Norway, Oslo, Norway; Department of Medical Epidemiology and Biostatistics, Karolinska Institutet, Stockholm, Sweden; Samfundet Folkhälsan, Helsinki, Finland; The Arctic University of Norway, Tromsø, Norway

**Keywords:** Premature death, Mortality, Modifiable risk factors, Attributable fraction, Absolute risk

## Abstract

**Background:**

Life expectancy is increasing in Europe, yet a substantial proportion of adults still die prematurely before the age of 70 years. We sought to estimate the joint and relative contributions of tobacco smoking, hypertension, obesity, physical inactivity, alcohol and poor diet towards risk of premature death.

**Methods:**

We analysed data from 264,906 European adults from the EPIC prospective cohort study, aged between 40 and 70 years at the time of recruitment. Flexible parametric survival models were used to model risk of death conditional on risk factors, and survival functions and attributable fractions (AF) for deaths prior to age 70 years were calculated based on the fitted models.

**Results:**

We identified 11,930 deaths which occurred before the age of 70. The AF for premature mortality for smoking was 31 % (95 % confidence interval (CI), 31–32 %) and 14 % (95 % CI, 12–16 %) for poor diet. Important contributions were also observed for overweight and obesity measured by waist-hip ratio (10 %; 95 % CI, 8–12 %) and high blood pressure (9 %; 95 % CI, 7–11 %). AFs for physical inactivity and excessive alcohol intake were 7 % and 4 %, respectively. Collectively, the AF for all six risk factors was 57 % (95 % CI, 55–59 %), being 35 % (95 % CI, 32–37 %) among never smokers and 74 % (95 % CI, 73–75 %) among current smokers.

**Conclusions:**

While smoking remains the predominant risk factor for premature death in Europe, poor diet, overweight and obesity, hypertension, physical inactivity, and excessive alcohol consumption also contribute substantially. Any attempt to minimise premature deaths will ultimately require all six factors to be addressed.

**Electronic supplementary material:**

The online version of this article (doi:10.1186/s12916-016-0630-6) contains supplementary material, which is available to authorized users.

## Background

Life expectancy in all countries of Western Europe has increased substantially over recent decades, primarily due to important decreases in mortality rates for death in middle age [1]. Within the European Union (EU27, 27 countries in the European Union), male age expectancy at age 40 ranges from 71 in the Baltic countries to around 80 in Mediterranean Europe, UK, and Sweden [2]. For women at age 40, the range is from 79 years in Bulgaria and Romania, to 86 years in France and Spain. If we define the age range 40–69 as ‘middle age’ and death occurring in this range as being premature, then about 20 % of men and 11 % of women in Europe (15 countries in the European Union, EU15) who reach the age of 40 can be expected to die prematurely based on current mortality rates [2].

Studies of premature death require the analysis of very large population cohorts or intervention studies with extensive baseline exposure information on major risk factors and complete mortality data. An alternative is to use a modelling approach, such as that taken by the Global Burden of Diseases (GBD) initiative, whereby estimates of exposure and disease risk from multiple sources are combined and used to approximate the contribution of different exposures to overall mortality and morbidity [1]. The GBD has estimated that, for Europe, the primary causes of premature mortality, in order of importance, are smoking, dietary risks, high blood pressure, high body mass index (BMI), physical inactivity, and high alcohol consumption. Other important factors are thought to include high cholesterol, high fasting plasma glucose, ambient air pollution, and occupational risks [3, 4]. While this initiative has resulted in extremely important information, and is the only option for most populations, it has a number of limitations such as the inclusion of data of variable quality from many different sources, and the inability to adjust comprehensively and consistently for other mortality risk factors that may confound the observed relationships. The GBD modelling approach is also usually not able to provide estimates of absolute risk and attributable fractions for important subgroups, e.g. for smokers and non-smokers separately, or for sets of risk factors combined.

To overcome these limitations, we have calculated estimates of the contribution of primary risk factors for premature death in Europe based on individual level data using the European Prospective Investigation into Cancer and Nutrition (EPIC) cohort study, which includes extensive exposure data on all primary risk factors, as well as complete follow-up for vital status, from 265,000 adults in 10 European countries.

## Methods

### Selection of risk factors

Our primary focus was on risk factors that are modifiable at a personal level, which have all been consistently associated with elevated risks of major chronic diseases and subsequent premature death, namely smoking [4–9], unhealthy diet [4, 10–12], high blood pressure [4, 9, 13], overweight and obesity [4, 14–18], physical inactivity [4, 6, 16, 19–22], and alcohol intake [23–27]. Additionally, we considered total cholesterol to HDL ratio (TC:HDL) [28, 29] and glycated haemoglobin (HbA1c) [4, 30–32].

### Study population

EPIC is an on-going multicentre prospective cohort study that recruited approximately 520,000 participants in 10 European countries (Denmark, France, Germany, Greece, Italy, The Netherlands, Norway, Spain, Sweden, and the United Kingdom) from 1992 to 2000. Approximately 70 % were women and most were aged between 35 and 70 years at baseline. A detailed description of the methods employed has previously been described [33, 34]. Blood samples were collected at baseline according to standardised procedures, and written informed consent for the baseline data collection and follow-up for vital status was provided by all study participants. For this analysis we excluded participants who were ≥ 75 or < 40 years old when recruited (*n* = 62,775); participants with missing questionnaire information (*n* = 30,048) or blood pressure measurements (*n* = 161,693); and participants with missing follow-up information (*n* = 1908). This analysis therefore included 264,906 participants (172,119 women and 92,787 men) from nine countries (as participants from Norway did not have their blood pressure measured). We also conducted sensitivity analyses including all participants with missing blood pressure to ensure that the estimates for the other covariates remained similar.

### Diet, lifestyle and anthropometric information

At recruitment, lifestyle and dietary questionnaires were used to obtain detailed information on all risk factors. Participants were classified as being never, former or current smokers at the time of interview. Diet and alcohol intake over the previous 12 months were assessed at study baseline using validated country-/centre-specific dietary questionnaires [33, 34], and alcohol intake was converted into grams of alcohol per day (g/day) by applying empirically derived definitions of standard drinks for each beverage and country. Both occupational and recreational physical activity were assessed via questionnaire using a validated scoring system [35].

Weight was measured with participants not wearing shoes to the nearest 0.1 kg; while height was measured – dependent on the study centre – to the nearest 0.1, 0.5, or 1.0 cm. Waist circumference was measured either at the smallest torso circumference or at the midpoint between the lower ribs and iliac crest. Hip circumference was measured horizontally at the level of the largest lateral extension of the hips or over the buttocks.

Systolic (SBP) and diastolic (DBP) blood pressure was measured on the right arms of participants while in sitting positions by trained personnel at baseline. Two separate readings were performed for each participant using a standard mercury manometer or oscillometric device, except in Denmark and Sweden, where one single measurement was taken in the supine position. To avoid any possible “white-coat” effect, if available, the second reading was used.

Finally, the EPIC Biomarkers sub-cohort of 16,775 randomly-selected participants (10,524 with complete risk factor data for the present analysis) was used to measure levels of circulating cholesterol (TC:HDL ratio) and HbA1c as a marker of average plasma glucose concentration. Total cholesterol and HDL-C levels were measured from serum (plasma for Umea, Sweden) using the Cobas® homogeneous enzymatic colorimetric test assay. HbA1c was measured from red blood cell fraction using the Tosoh (HLC-723G8) ion exchange high-performance liquid chromatography assay.

### Assessment of mortality

Data on vital status and the cause and date of death were collected at the EPIC study centres using record linkages with cancer registries, boards of health and death indices, or through active follow-up. End of follow-up was defined as the latest date of complete follow-up for vital status, which was between 2008 and 2010 dependent on study centre.

### Definition of premature mortality

We provide estimates of the contribution for various risk factors to the risk of dying before 70 years of age, conditional on surviving to age 40 years. We additionally provide these estimates for death prior to ages 65 and 75 years in Additional file 1: Table S3 and Additional file 2: Table S4.

### Statistical methods

Participants were classified as never, former, or current smokers. Alcohol consumption was modelled using the following categories (standard drinks/day, with a standard drink defined as 10 g of alcohol): 0, 0–0.5, 0.5–1, 1–2, 2–6, 6–10, and > 12 (men only). The dietary score used was an adapted version of the WCRF/AICR score [11], including the intakes of (1) energy dense foods/sugary drinks, (2) plant foods (fruits/vegetables/dietary fibre), and (3) animal foods (red and processed meat). The derived dietary score was categorised into four groups: unhealthy, moderately unhealthy, moderately healthy, and healthy. Full details of dietary score derivation can be found in the Additional file 3: Methods. Occupational and recreational physical activity were combined and categorised into four groups (“The Cambridge Index”) – inactive, moderately inactive, moderately active and active. BMI was calculated as kg/m^2^ and categorised as < 20, 20–21.9, 22–24.9 (reference), 25–29.9, 30–34.9, and ≥ 35. We constructed five categories of waist-hip ratio (WHR) by splitting the distribution at its sex-specific quintiles. Blood pressure was categorised into clinical cut-points: normal (SBP < 120 mm Hg and DBP < 80 mm Hg); pre-hypertension (SBP ≥ 120 mm Hg or DBP ≥ 80 mm Hg); hypertension 1 (SBP ≥ 140 mm Hg or DBP ≥ 90 mm Hg); and hypertension 2 (SBP ≥ 160 mm Hg or DBP ≥ 100 mm Hg) [36]. Sex-specific fourths were created for both TC:HDL and HbA1c.

Hazard ratios (HR) and 95 % confidence intervals (CI) for all-cause mortality were estimated using flexible parametric survival models on the cumulative hazards scale [37, 38], which – in addition to the HR – allow direct estimation of the conditional cumulative hazard function, and thus absolute risks of death. Within these models we employed restricted cubic splines with three internal knots to model the baseline hazard using attained age as the time-scale. Separate models were fitted for men and women, as well as for both sexes combined. All models included age at baseline, smoking status, dietary score, alcohol intake, physical activity, blood pressure, and either BMI or WHR. Finally, we considered models that also included the TC:HDL ratio or HbA1c in the sub-cohort which had these measures available. We present HRs and CIs for premature mortality estimated using models fit to follow-up data censored at 70 years of age. We investigated non-proportional hazards by fitting interactions between covariates and the time-scale (attained age), and the final models allowed the parameters for smoking status to vary over time. We also examined whether there were any important interactions between pairs of covariates by comparing models with and without interaction terms using the likelihood-ratio test.

Model-based survival functions and their CI were obtained from fitted models at specific combinations of covariate values. These survival functions were conditional on surviving until age 40 years. We also calculated attributable fractions (AFs) for each covariate based on the predicted survival functions evaluated at age 70. These AFs rely on the comparison between the expected survival at age 70 under the following scenarios: (1) the observed distribution of risk factors in the cohort and (2) an alternative, reference (counterfactual) distribution of risk factors corresponding to the removal of a specific risk factor or set of risk factors. For most risk factors, the reference distribution was simply set so each member of the cohort was “unexposed” to the risk factor, or in the category of the risk factor associated with the lowest risk of death. These attributable risks thus represent a “best-case”, in that they are calculated based on a hypothetical reference population with risk factors removed entirely. For smoking, the reference distribution was a cohort of never smokers. For most risk factors the reference distribution was similarly defined, i.e. normal blood pressure, most healthy diet, physically active, and lowest category of WHR. For BMI, the reference distribution was set to 22–25 for all participants with a BMI above 22, and left unchanged for those with a BMI < 22. The reference distribution for alcohol intake was a population that drinks no more than one or two standard drinks per day. Thus, the reference distribution removes the excess risk associated with drinking more than two drinks per day, without removing the apparent excess risk from consuming less than one drink per day or abstaining. See Additional file 3: Methods for technical details of the AF calculations. All statistical analyses were conducted using Stata version 12.1 and R version 3.1.2.

## Results

Among the 264,906 EPIC participants (172,119 women and 92,787 men) with complete baseline and follow-up data who were included in this analysis, we identified 11,930 premature deaths (i.e. before the age of 70) during a median follow-up of 11.5 years. These deaths were predominantly due to cancer (5907, 50 %) and circulatory diseases (2580, 22 %). Survival functions for men and women in our study sample are presented in Additional file 4: Figure S1. Survival to age 70 was somewhat higher in EPIC (93 % for women and 86 % for men) than that expected based on the general European population (89 % for women and 80 % for men, calculated based on mortality rates from the EU15) [2]. After accounting for sex and smoking status, the survival functions were similar across all countries, with the exception of Italy and France, which had higher survival in each of the sex/smoking strata (Additional file 5: Figure S2). The distributions of the risk factors are presented in Table 1. The number of participants by country and sex are presented in Additional file 6: Table S1 and the distribution of covariates by country is presented in Additional file 7: Table S2.

**Table 1:** Baseline and covariate distributions in the EPIC cohort: overall, for premature deaths (prior to age 70 years), and by sex

	All					Sex			
		Total		Premature deaths		Male		Female	
		n = 264,906	%	*n* = 11,930	%	*n* = 92,787	%	*n* = 172,119	%
Age at baseline, years	40–50	67,229	25	1597	13	21,270	23	45,959	27
	50–60	124,557	47	7302	61	44,441	48	80,116	47
	60–70	64,640	24	3031	25	23,492	25	41,148	24
Smoking	Never smoker	121,743	46	3365	28	26,462	29	95,281	55
	Former smoker	79,391	30	3489	29	37,967	41	41,424	24
	Current smoker	63,772	24	5076	43	28,358	31	35,414	21
Blood pressure	Normal	54,935	21	1793	15	11,253	12	43,682	25
	Pre-hypertension	102,263	39	4023	34	35,844	39	66,419	39
	Hypertension 1	72,429	27	3711	31	30,481	33	41,948	24
	Hypertension 2	35,279	13	2403	20	15,209	16	20,070	12
BMI (kg/m^2^)	< 20	12,001	5	665	6	1447	2	10,554	6
	20–21.9	30,376	11	1244	10	5443	6	24,933	14
	22–24.9 (reference)	77,680	29	3127	26	24,984	27	52,696	31
	25–29.9 (overweight)	103,379	39	4602	39	46,392	50	56,987	33
	30–34.9 (obese)	32,270	12	1672	14	12,391	13	19,879	12
	35+ (very obese)	9200	3	620	5	2130	2	7070	4
Waist-to-hip ratio (sex-specific fifths)	1	57,336	22	1960	16	19,000	20	38,336	22
	2	52,688	20	2102	18	21,985	24	30,703	18
	3	59,966	23	2372	20	18,517	20	41,449	24
	4	45,066	17	2200	18	14,844	16	30,222	18
	5	49,850	19	3296	28	18,441	20	31,409	18
Alcohol intake (drinks/day)	0	32,826	12	1598	13	5941	6	26,885	16
	0–0.5	78,871	30	3054	26	16,748	18	62,123	36
	0.5–1	42,158	16	1580	13	13,255	14	28,903	17
	1–2	50,904	19	2073	17	20,550	22	30,354	18
	2–6	52,468	20	2741	23	29,860	32	22,608	13
	> 6 (women), 6–10 (men)	6683	3	690	6	5437	6	1246	1
	> 10 (men)	996	0	194	2	996	1	0	0
Diet	Unhealthy	22,346	8	1746	15	12,617	14	9729	6
	Moderately unhealthy	112,965	43	5792	49	48,238	52	64,727	38
	Moderately healthy	77,110	29	2800	23	20,515	22	56,595	33
	Healthy	52,485	20	1592	13	11,417	12	41,068	24
Physical activity	Inactive	61,771	23	3155	26	18,658	20	43,113	25
	Moderately inactive	88,907	34	3753	31	29,062	31	59,845	35
	Moderately active	60,548	23	2531	21	22,015	24	38,533	22
	Active	53,680	20	2491	21	23,052	25	30,628	18
	All	10,524	100	469	100	3899	100	6625	100
Total to HDL cholesterol ratio (sex-specific fourths)	< 3.79 (men), < 3.09 (women)	2530	24	73	16	933	24	1597	24
	3.79–4.64 (men), 3.09–3.76 (women)	2531	24	96	20	921	24	1610	24
	4.64–5.73 (men), 3.76–4.69 (women)	2517	24	118	25	920	24	1597	24
	5.73 + (men), 4.69 + (women)	2503	24	159	34	923	24	1580	24
	missing	443	4	23	5	202	5	241	4
Glycated haemoglobin (%) (sex-specific fourths)	< 5.26	3121	30	120	26	1154	30	1967	30
	5.26–5.54 (men), 5.26–5.44 (women)	2543	24	100	21	1156	30	1387	21
	5.54–5.72 (men), 5.44–5.72 (women)	2354	22	96	20	619	16	1735	26
	5.72 +	2399	23	142	30	934	24	1465	22
	missing	107	1	11	2	36	1	71	1

### Hazard ratios

The HRs for premature mortality are presented in Table 2. After mutual adjustment for all risk factors, over a two-fold greater risk was observed for current smokers when compared with never smokers for both men (HR, 2.54; 95 % CI, 2.36–2.73) and women (HR, 2.14; 95 % CI, 2.01–2.29), with former smokers having an intermediate risk. When compared with the physically inactive group, being physically very active was associated with substantially lower mortality rates for men (HR, 0.73; 95 % CI, 0.68–0.78) and women (HR, 0.69; 95 % CI, 0.64–0.76). Men and women with a ‘healthy’ diet had 22 % (HR, 0.78; 95 % CI, 0.69–0.88) and 25 % (HR, 0.75; 95 % CI, 0.67–0.83) lower mortality rates, respectively, when compared with the ‘unhealthy’ diet groups. J-shaped relationships between alcohol consumption at baseline and mortality were observed for men and women, with the highest mortality rates found for the highest consumers (>6 drinks/day for women; HR, 1.63; 95 % CI, 1.31–2.04 and > 10 drinks/day for men; HR, 2.38; 95 % CI, 2.02–2.79) when compared with the moderate alcohol consumption reference group (1–2 drinks per day). For BMI (unadjusted for WHR), when compared with the 22–24.9 reference group, higher mortality rates were observed for the extreme high (35+: men, HR, 1.77; 95 % CI, 1.50–2.09; women, HR, 1.40; 95 % CI, 1.20–1.63) and low (<20: men, HR, 1.76; 95 % CI, 1.54–2.01; women, HR, 1.55; 95 % CI, 1.38–1.75) BMI groups. For WHR (unadjusted for BMI), when the highest and lowest fifths were compared, 39 % (HR, 1.39; 95 % CI, 1.28–1.51) and 52 % (HR, 1.52; 95 % CI, 1.39–1.65) higher premature mortality rates were observed for women and men, respectively. Participants with hypertension also had higher premature mortality rates than those with normal blood pressure levels, with an increasing gradient for hypertension level 1 (HR, 1.17; 95 % CI, 1.09–1.25) and level 2 (HR, 1.52; 95 % CI, 1.40–1.64).

**Table 2:** Hazard ratios and confidence intervals for death prior to age 70 years in the EPIC cohort

		Overall				Women				Men			
		Model 1		Model 2		Model 1		Model 2		Model 1		Model 2	
		HR	(95 % CI)	HR	(95 % CI)	HR	(95 % CI)	HR	(95 % CI)	HR	(95 % CI)	HR	(95 % CI)
Smoking status	Never	1.00		1.00		1.00		1.00		1.00		1.00	
	Former	1.39	(1.32–1.46)	1.37	(1.30–1.43)	1.30	(1.21–1.39)	1.29	(1.20–1.38)	1.47	(1.36–1.58)	1.43	(1.32–1.54)
	Smoker	2.38	(2.27–2.50)	2.36	(2.25–2.48)	2.16	(2.03–2.30)	2.14	(2.01–2.29)	2.57	(2.39–2.77)	2.54	(2.36–2.73)
Physical activity	Inactive	1.00		1.00		1.00		1.00		1.00		1.00	
	Moderately inactive	0.78	(0.74–0.82)	0.79	(0.75–0.82)	0.78	(0.73–0.84)	0.79	(0.73–0.84)	0.77	(0.72–0.82)	0.77	(0.72–0.83)
	Moderately active	0.75	(0.71–0.79)	0.75	(0.71–0.79)	0.72	(0.66–0.78)	0.72	(0.67–0.78)	0.76	(0.70–0.82)	0.77	(0.71–0.83)
	Active	0.71	(0.67–0.75)	0.72	(0.68–0.76)	0.69	(0.64–0.75)	0.69	(0.64–0.76)	0.72	(0.67–0.77)	0.73	(0.68–0.78)
Diet	Unhealthy	1.00		1.00		1.00		1.00		1.00		1.00	
	Moderately unhealthy	0.87	(0.82–0.92)	0.88	(0.83–0.93)	0.85	(0.77–0.93)	0.85	(0.77–0.93)	0.88	(0.82–0.94)	0.89	(0.83–0.95)
	Moderately healthy	0.80	(0.75–0.85)	0.81	(0.76–0.87)	0.78	(0.70–0.86)	0.79	(0.71–0.87)	0.80	(0.74–0.88)	0.82	(0.75–0.90)
	Healthy	0.75	(0.70–0.81)	0.77	(0.72–0.83)	0.73	(0.66–0.82)	0.75	(0.67–0.83)	0.76	(0.67–0.85)	0.78	(0.69–0.88)
Alcohol intake (drinks/day)	0	1.58	(1.47–1.70)	1.58	(1.47–1.70)	1.46	(1.33–1.61)	1.46	(1.32–1.60)	1.90	(1.70–2.12)	1.91	(1.71–2.14)
	0–0.5	1.17	(1.10–1.25)	1.18	(1.11–1.25)	1.13	(1.04–1.22)	1.13	(1.04–1.22)	1.26	(1.14–1.38)	1.27	(1.15–1.39)
	0.5–1	1.00		1.00		1.00		1.00		1.00		1.00	
	1–2	0.97	(0.91–1.04)	0.97	(0.91–1.04)	0.98	(0.90–1.08)	0.98	(0.89–1.08)	0.97	(0.89–1.07)	0.97	(0.88–1.07)
	2–6	1.07	(1.00–1.14)	1.06	(1.00–1.13)	1.16	(1.05–1.27)	1.15	(1.04–1.26)	1.04	(0.96–1.14)	1.03	(0.95–1.13)
	> 6 (women), 6–10 (men)	1.51	(1.38–1.66)	1.47	(1.34–1.62)	1.72	(1.38–2.15)	1.63	(1.31–2.04)	1.48	(1.32–1.65)	1.45	(1.29–1.62)
	> 10 (men)	2.47	(2.12–2.88)	2.40	(2.06–2.79)					2.43	(2.07–2.85)	2.38	(2.02–2.79)
Blood pressure	Normal	1.00		1.00		1.00		1.00		1.00		1.00	
	Pre-hypertension	1.03	(0.97–1.09)	0.99	(0.93–1.04)	1.04	(0.96–1.11)	1.00	(0.93–1.07)	1.04	(0.95–1.14)	0.99	(0.90–1.08)
	Hypertension 1	1.19	(1.12–1.26)	1.12	(1.06–1.19)	1.16	(1.07–1.26)	1.10	(1.02–1.19)	1.24	(1.13–1.37)	1.15	(1.05–1.27)
	Hypertension. 2	1.56	(1.46–1.67)	1.45	(1.36–1.55)	1.50	(1.37–1.65)	1.42	(1.29–1.55)	1.63	(1.47–1.81)	1.50	(1.36–1.66)
BMI (kg/m^2^)	< 20	1.62	(1.48–1.76)			1.47	(1.33–1.63)			1.98	(1.70–2.30)		
	20–21.9	1.17	(1.09–1.25)			1.10	(1.01–1.20)			1.25	(1.13–1.40)		
	22–24.9 (reference)	1.00				1.00				1.00			
	25–29.9 (overweight)	0.98	(0.94–1.03)			1.03	(0.96–1.10)			0.97	(0.91–1.03)		
	30–34.9 (obese)	1.16	(1.09–1.23)			1.05	(0.95–1.15)			1.26	(1.16–1.37)		
	35+ (very obese)	1.61	(1.47–1.76)			1.55	(1.38–1.75)			1.76	(1.54–2.01)		
Waist-to-hip ratio (fifths)	1			1.00				1.00				1.00	
	2			1.01	(0.95–1.07)			1.00	(0.91–1.09)			1.03	(0.95–1.13)
	3			1.02	(0.96–1.08)			0.99	(0.92–1.08)			1.06	(0.97–1.15)
	4			1.19	(1.11–1.26)			1.15	(1.05–1.25)			1.24	(1.13–1.36)
	5			1.44	(1.36–1.53)			1.39	(1.28–1.51)			1.52	(1.39–1.65)

Estimates from flexible parametric survival models with attained age as the time-scale. Models included all listed covariates, as well as age at baseline attendance and country of recruitment. Overall estimates are also adjusted for sex. Model 1 includes BMI, whereas Model 2 includes waist-to-hip ratio as a measure of adiposity. Model 1 and Model 2 are otherwise identical

### Attributable fractions

Attributable fractions (AF) for premature mortality were calculated overall and by sex, and also for never smokers and current smokers separately (Table 3). Given the monotonic association between mortality and WHR, this was used as the primary measure of overweight and obesity, although all results were calculated using BMI also. The AF calculations represent “best-case” estimates based on the hypothetical removal of risk-elevating factors from the population entirely.

**Table 3:** Population attributable fractions of deaths prior to age 70 given the distribution of covariates in the EPIC cohort, using waist-to-hip ratio to assess for obesity

		All participants		Never smokers		Current smokers	
	Covariate^a^	Overall^b^	Cumulative^c^	Overall^b^	Cumulative^c^	Overall^b^	Cumulative^c^
Women and Men	Smoking	0.31 (0.31–0.32)	0.31 (0.31–0.32)	–	–	0.56 (0.55–0.56)	0.56 (0.55–0.56)
	Diet	0.14 (0.12–0.16)	0.41 (0.39–0.43)	0.12 (0.10–0.14)	0.12 (0.10–0.14)	0.16 (0.13–0.18)	0.63 (0.62–0.64)
	Overweight and obesity (WHR)	0.10 (0.08–0.12)	0.47 (0.45–0.49)	0.10 (0.08–0.11)	0.21 (0.18–0.23)	0.10 (0.08–0.12)	0.67 (0.66–0.68)
	High blood pressure	0.09 (0.07–0.11)	0.52 (0.50–0.54)	0.09 (0.08–0.11)	0.28 (0.25–0.31)	0.08 (0.07–0.10)	0.70 (0.69–0.71)
	Physical inactivity	0.07 (0.05–0.09)	0.56 (0.54–0.57)	0.08 (0.06–0.10)	0.34 (0.31–0.36)	0.07 (0.05–0.09)	0.72 (0.71–0.74)
	Alcohol intake	0.04 (0.03–0.04)	0.57 (0.55–0.59)	0.02 (0.01–0.02)	0.35 (0.32–0.37)	0.05 (0.04–0.06)	0.74 (0.73–0.75)
	Combined	0.57 (0.55–0.59)		0.35 (0.32–0.37)		0.74 (0.73–0.75)	
Women	Smoking	0.26 (0.25–0.26)	0.26 (0.25–0.26)	–	–	0.55 (0.54–0.55)	0.55 (0.54–0.55)
	Diet	0.14 (0.11–0.16)	0.36 (0.33–0.38)	0.12 (0.09–0.15)	0.12 (0.09–0.15)	0.16 (0.13–0.19)	0.62 (0.60–0.63)
	Overweight and obesity (WHR)	0.07 (0.05–0.09)	0.40 (0.38–0.43)	0.07 (0.05–0.09)	0.18 (0.15–0.22)	0.07 (0.05–0.10)	0.65 (0.63–0.66)
	High blood pressure	0.10 (0.07–0.12)	0.46 (0.43–0.48)	0.11 (0.08–0.13)	0.27 (0.23–0.30)	0.09 (0.07–0.11)	0.68 (0.66–0.70)
	Physical inactivity	0.06 (0.03–0.09)	0.49 (0.46–0.52)	0.07 (0.03–0.10)	0.32 (0.28–0.35)	0.06 (0.03–0.09)	0.70 (0.68–0.72)
	Alcohol intake	0.02 (0.01–0.03)	0.50 (0.47–0.53)	0.01 (0.01–0.02)	0.32 (0.28–0.36)	0.03 (0.02–0.04)	0.71 (0.69–0.72)
	Combined	0.50 (0.47–0.53)		0.32 (0.28–0.36)		0.71 (0.69–0.72)	
Men	Smoking	0.37 (0.35–0.38)	0.37 (0.35–0.38)	–	–	0.57 (0.56–0.57)	0.57 (0.56–0.57)
	Diet	0.13 (0.09–0.17)	0.45 (0.43–0.48)	0.13 (0.09–0.17)	0.13 (0.09–0.17)	0.14 (0.10–0.18)	0.63 (0.61–0.65)
	Overweight and obesity (WHR)	0.13 (0.11–0.16)	0.53 (0.50–0.55)	0.12 (0.09–0.14)	0.23 (0.19–0.27)	0.13 (0.11–0.16)	0.69 (0.67–0.70)
	High blood pressure	0.08 (0.05–0.12)	0.57 (0.54–0.60)	0.08 (0.05–0.12)	0.29 (0.25–0.34)	0.07 (0.04–0.11)	0.71 (0.69–0.73)
	Physical inactivity	0.07 (0.05–0.10)	0.60 (0.57–0.63)	0.07 (0.05–0.10)	0.35 (0.30–0.39)	0.07 (0.05–0.10)	0.74 (0.72–0.75)
	Alcohol intake	0.06 (0.05–0.07)	0.63 (0.60–0.65)	0.04 (0.02–0.05)	0.37 (0.32–0.41)	0.07 (0.06–0.09)	0.76 (0.74–0.77)
	Combined	0.63 (0.60–0.65)		0.37 (0.32–0.41)		0.76 (0.74–0.77)	

^a^Attributable fractions were calculated based on the difference in expected cumulative risk given the observed covariate distributions in EPIC and the expected cumulative risk under the following scenarios. Smoking: A population of never smokers. Diet: A population of people in the healthy category. Blood pressure: A population of people with normal blood pressure. High alcohol intake: A population who drink at most 1–2 drinks per day. Physical activity: A population of people in the active category. Overweight and obesity: A population of people with WHR below the lowest sex-specific quintile. These attributable risks thus represent a “best-case”, in that they are calculated based on a hypothetical reference population with risk factors removed entirely

^b^Estimated using predictions from a model mutually adjusted for all listed covariates as well as age at baseline. Attributable fractions are based on modifying one covariate at a time, with the distribution of the remaining covariates left as observed in EPIC

^c^The cumulative attributable fraction after the sequential addition of each covariate

The AF for smoking was 31 % (26 % among women, 37 % among men, and 56 % among current smokers overall; Table 3). For diet and WHR the AF was 14 % and 10 %, respectively. Overall, 9 % of premature deaths were attributed to high blood pressure, and the AFs for physical inactivity and high alcohol intake were 7 % and 4 %, respectively. The role of alcohol was more important among men than women (6 % vs. 2 %). When BMI was used instead of WHR, the effect of being overweight/obese was less apparent, with the AF estimated to be 3 % (Additional file 1: Table S3).

Because these risk factors co-vary to a large degree, it is not possible to sum up the AF estimates to come to an overall estimate. We can derive such an estimate by removing the effect of each risk factor consecutively, yielding a cumulative AF for a combination of risk factors. After accounting for the fraction of premature deaths attributable to smoking, an additional 10 % can be attributed to poor diet. Beyond this, an additional 6 % were attributed to overweight and obesity (WHR), and 5 % to high blood pressure (Table 3). Overall, 52 % of all premature deaths can be attributed to these four factors. The two remaining factors (physical activity and alcohol intake) added an additional 5 %, resulting in a total AF of 57 %. The AF for premature mortality for all six exposures was 74 % among current smokers (56 % smoking) and 35 % among never smokers. The attributable fractions were similar for deaths prior to age 65 (Additional file 2: Table S4) and 75 years (Additional file 8: Table S5), and remained similar in sensitivity analyses including all participants with missing blood pressure (Additional file 9: Table S6).

### Survival curves

**Fig. 1:**
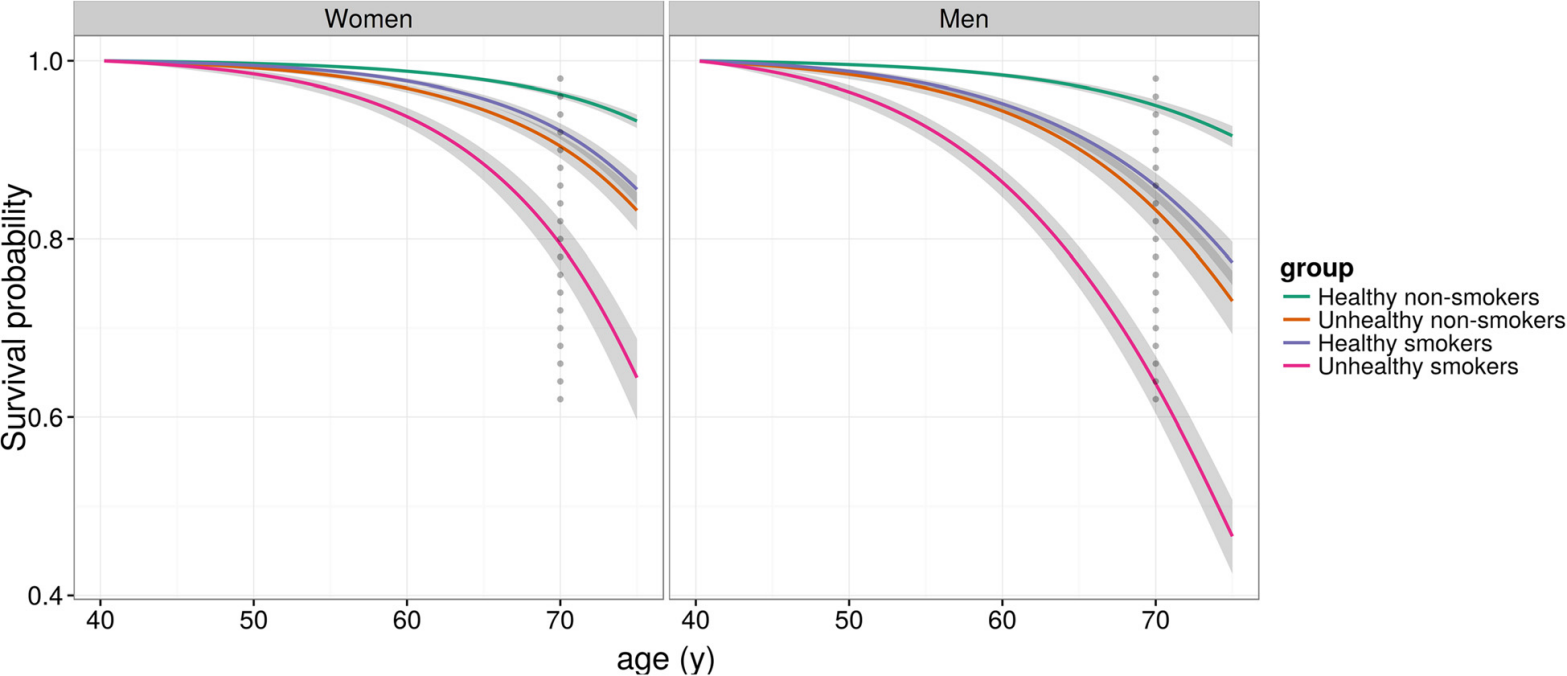
Model-based survival curves by smoking status and other individual factors. “Healthy” participants are those with a body mass index (BMI) of 22–25 who are moderately active, have normal blood pressure, eat a healthy diet, and drink one to two drinks per day. “Unhealthy” participants are those with a BMI of 30–35 who are inactive, hypertensive, eat an unhealthy diet, and consume more than two drinks per day

To estimate the effect that these risk factors can have on expected survival at an individual level, we compared survival curves for current smokers and non-smokers after further stratifying by whether they had otherwise “healthy” or “unhealthy” characteristics (Fig. 1). ‘Healthy’ was defined as a BMI of 22–25, having normal blood pressure, being moderately physically active, eating a healthy diet, and drinking one to two drinks per day. “Unhealthy” was defined as a BMI of 30–35, being physically inactive, eating an unhealthy diet, being hypertensive, and consuming more than two drinks per day. Comparison of the four groups indicated that 96 % of “healthy” non-smoking women (95 % CI, 96–97 %) and 95 % of “healthy” non-smoking men (95 % CI, 94–96 %) could be expected to survive to 70. Conversely, only 64 % (95 % CI, 60–67 %) and 79 % (95 % CI, 76–82 %) of smoking men and women with additional unhealthy characteristics, respectively, could be expected to live to this age. The two intermediate groups, smokers with otherwise healthy characteristics and ‘unhealthy’ non-smokers, had similar expected survival.

### Cholesterol and glycated haemoglobin

Based on the EPIC-Biomarkers sub-cohort of 10,524 individuals, including 469 premature deaths, an increased risk of premature mortality was seen across all four categories of TC:HDL (Additional file 10: Table S7), reaching a 63 % increase in the fourth (highest) compared with the first (lowest) quartile. Based on this modest sample size, the AF for TC:HDL was estimated to be 15 % (95 % CI, 7–23 %). Conversely, no apparent increase in risk was observed for higher levels of HbA1c.

## Discussion

Our analysis of the primary causes of premature death among more than 250,000 European adults indicates that the four major risk factors are tobacco smoking, poor diet, obesity, and high blood pressure, which together account for over 50 % of premature deaths. Two other risk factors, physical inactivity and excessive alcohol consumption, have AFs of 7 % and 4 % of premature deaths, respectively. Our study also provides preliminary evidence for an important AF for high cholesterol levels, although the sample size was limited.

The GBD has derived similar estimates for the role of each exposure using an alternative modelling approach, relying largely on published estimates of effect for each exposure and estimates of exposure prevalence in each population [1]. Their estimates of AFs for premature mortality for the European population provide broadly comparable estimates for smoking, poor diet and high blood pressure (Table 4). However, the GBD estimates are approximately twice as high for excessive alcohol consumption (8 % vs. 4 %), and substantially higher for excessive body mass (14 % vs. 3 % based on BMI). Using WHR as a measure of obesity made the estimates more comparable (10 % based on WHR).

**Table 4:** Comparison of population attributable fractions (%, 95 % CI) from the Global Burden of Diseases analysis with those from the present EPIC analysis

	GBD	EPIC
Tobacco smoking	25 (22–27)	31 (31–32)
Dietary risks	23 (21–26)	14 (12–16)
High blood pressure	15 (13–17)	9 (7–11)
High body mass index	14 (12–15)	3 (2–5)
High waist-to-hip ratio	–	10 (8–12)
Physical inactivity and low physical activity	9 (8–11)	7 (5–9)
High alcohol use	8 (7–9)	4 (3–4)

Estimates from the GBD are taken from the website http://vizhub.healthdata.org/gbd-compare. They are the estimated attributable fractions for death in Western Europe for the age range 50–69 years for each risk factor

There are three possible explanations for these differences. Firstly, the estimates of relative risk used in the calculations might differ – indeed, we estimated modest relative risks for overweight and obesity and physical inactivity. Secondly, the distribution of the risk factors used for the GBD computations might differ from the distribution in EPIC which, for example, includes relatively few very heavy consumers of alcohol or very obese participants. This is a well-known phenomenon in prospective cohort studies, also called “healthy volunteer” effect. Finally, the reference or counterfactual distributions used for the AF calculations might differ. For instance, the GBD used a “theoretical minimum-risk exposure distribution”. On the other hand, we have chosen to not necessarily use a theoretically “optimal” exposure distribution in all cases. For instance, using the lowest risk category for alcohol intake or BMI would involve an increase in alcohol intake or BMI in a proportion of the participants. Instead, we have focused on the AF for high alcohol intake and overweight and obesity per se. Low reported alcohol intake in particular is associated with substantially higher risk of death in EPIC, possibly due to the influence of former drinkers who quit for health reasons or misclassification of heavy drinkers [23, 27, 39]. This misclassification would lead to an underestimation of the AF for alcohol in the EPIC study, and – assuming that the GBD estimates do not suffer from the same problems – may explain the difference between the EPIC and GBD estimates in this case.

On an individual level, the estimated conditional survival curves suggest smoking could have a similar effect on survival to age 70 to that of all other factors combined. Men who were smokers but possessed otherwise healthy characteristics had expected survival of 86 %, similar to the 83 % expected survival for men with unhealthy characteristics but who never smoked. For women, both smokers with otherwise healthy characteristics and unhealthy non-smokers also had similar expected survival (92 % and 90 %, respectively). These estimates reinforce the critical importance of smoking in terms of preventing premature death, and suggest it is as important as the other five major risk factors combined.

A strength of our approach is the ability to estimate AFs for important sub-groups, such as smokers and never smokers, and also the direct estimation of AFs for combinations of risk allowing for their interdependence. Interestingly, our results indicate that, for both current and never smokers, an equivalent proportion of premature deaths can be attributed to poor diet, hypertension, overweight and obesity, and physically inactivity.

The principal limitation of our study is the relatively small number of participants with available cholesterol and HbA1c measurements, leading to imprecise estimates of relative risks, prevalence, and AF for these factors. Further, given that all exposures were assessed only once at recruitment to the study, we could not assess the potential effects of changing exposures over time, such as quitting smoking, gaining or losing weight, or increasing physical activity. As such, our estimated AFs and survival functions cannot be interpreted as the expected effects on mortality if individuals were to change their lifestyle or diet, but rather reflect comparisons of individuals with a given, constant pattern of exposures, or hypothetical scenarios in which no-one in the population is exposed to a given risk factor. Similarly, with only one assessment of exposure we cannot assess the potential effects of measurement error, which are unlikely to be equal across the six factors (e.g. BMI and blood pressure are subject to only modest measurement error, especially compared with self-reported diet and physical activity), and can lead to under- or overestimates of the risk associated with specific risk factors. Finally, the participants in EPIC are not representative of the general European population and may have a different distribution of risk factors than other target populations. In addition to the participants not being a representative sample of any population at baseline, during the follow-up period, the participants have aged, so estimates presented here do not strictly represent the age group 40 to 70.

We choose to include individuals who reported a prevalent chronic disease condition at baseline (e.g. diabetes, heart disease, angina, or a previous diagnosis of cancer), as such a sample would be more representative of the underlying population. Excluding the 7 % of the cohort who did report a prevalent chronic disease at baseline did not have any substantial effect on our results. As an additional sensitivity analysis to evaluate the possibility of sick individuals changing their lifestyle habits relatively recently prior to interview, we also excluded the first 4 years of follow-up. Again, this exclusion did not substantially affect the results.

## Conclusions

In summary, we used individual level data from a large European prospective cohort study to estimate the relative contributions of various factors to premature mortality both on the population level and the individual level. While smoking remains the predominant risk factor for premature mortality in Europe, poor diet, obesity, and hypertension also have a substantial additional effect. We also provided an estimate of the incidence of death prior to age 70 years that could be expected among an otherwise healthy non-smoking population (about 4 %). Our results indicate that it is of public health importance to persist and extend the fight against smoking as well as to promote healthy behaviour, including better diet, avoidance of overweight/obesity, physical activity, and blood pressure and blood lipid control with the aim of minimising premature mortality.

## Abbreviations

AF, attributable fraction; BMI, body mass index; CI, confidence interval; DBP, diastolic blood pressure; EPIC, European Prospective Investigation into Cancer and Nutrition; GBD, Global Burden of Diseases; HR, hazard ratio; SBP, systolic blood pressure; WHR, waist-to-hip ratio

## Additional files

Additional file 1: Table S3. Population attributable fractions of deaths prior to age 65 years given the distribution of covariates in the EPIC cohort, using waist-to-hip ratio to assess for obesity. (PDF 75 kb) Additional file 2: Table S4. Population attributable fractions of deaths prior to age 75 years given the distribution of covariates in the EPIC cohort, using waist-to-hip ratio to assess for obesity. (PDF 76 kb) Additional file 3: Methods 1. Supplementary Methods for “Modifiable causes of death in middle-age in Western Europe: results from the EPIC cohort study”. (PDF 100 kb) Additional file 4: Figure S1. Model-based survival functions for men and women in EPIC (coloured lines) and a random sample of Kaplan–Meier estimates of the survival function evaluated at failure times (grey dots). Survival to age 70 years in the EU in general was estimated to be 0.89 for women and 0.80 for men (using mortality rates from 2006–2010 obtained from http://ec.europa.eu/eurostat). (TIFF 402 kb) Additional file 5: Model -based survival functions for each country in EPIC, by sex and smoking status. (TIFF 824 kb) Additional file 6: Table S1. Number of participants in the EPIC cohort: overall, for premature deaths (prior to age 70 years), by country of recruitment and sex. (PDF 32 kb) Additional file 7: Table S2. Attributable fractions of deaths prior to age 70 years given the distribution of covariates in the EPIC cohort, using body mass index to assess overweight and obesity. (PDF 49 kb) Additional file 8: Table S5. Hazard ratios and confidence intervals for death prior to age 70 years in the EPIC cohort. (PDF 76 kb) Additional file 9: Table S6. Sensitivity analysis including all participants with missing blood pressure. Population attributable fractions of deaths prior to age 70 years given the distribution of covariates in the EPIC cohort, using waist-to-hip ratio to assess for obesity. (PDF 75 kb) Additional file 10: Table S7. Hazard ratios and confidence intervals for death prior to age 70 years in the EPIC cohort. (PDF 42 kb)
