# Supplementary material for: Modifiable causes of premature death in middle-age in Western Europe: results from the EPIC cohort study
Source: BMC Med. 2016 Jun 14;14:87. doi: 10.1186/s12916-016-0630-6 (PMC4907105; doi:10.1186/s12916-016-0630-6)
Supplement: Additional file 1: Table S3. — Population attributable fractions of deaths prior to age 65 years given the distribution of covariates in the EPIC cohort, using waist-to-hip ratio to assess for obesity. (PDF 75 kb) [file 12916_2016_630_MOESM1_ESM.pdf]

S3 Table. attributable fractions of deaths prior to age 70 given the distribution of covariates in the EPIC cohort, using BMI to assess overweight and obesity.

		All participants		Never-smokers		Current-smokers	
	Covariate[1]	Overall[2]	Cumulative[3]	Overall[2]	Cumulative[3]	Overall[2]	Cumulative[3]
Women and Men	Smoking	0.32 [0.31,0.33]	0.32 [0.31,0.33]	-	-	0.57 [0.56,0.57]	0.57 [0.56,0.57]
	Diet	0.14 [0.12,0.17]	0.42 [0.40,0.43]	0.13 [0.10,0.15]	0.13 [0.10,0.15]	0.16 [0.13,0.18]	0.64 [0.63,0.65]
	High blood pressure	0.12 [0.11,0.14]	0.49 [0.48,0.51]	0.13 [0.11,0.14]	0.24 [0.21,0.26]	0.11 [0.10,0.13]	0.68 [0.67,0.70]
	Physical inactivity	0.07 [0.05,0.09]	0.53 [0.51,0.55]	0.08 [0.06,0.10]	0.30 [0.27,0.33]	0.07 [0.05,0.09]	0.71 [0.70,0.72]
	Alcohol intake	0.04 [0.04,0.05]	0.55 [0.53,0.57]	0.02 [0.01,0.02]	0.31 [0.28,0.34]	0.06 [0.05,0.07]	0.73 [0.71,0.74]
	Overweight and obesity (BMI)	0.03 [0.02,0.05]	0.56 [0.54,0.58]	0.04 [0.03,0.05]	0.34 [0.31,0.36]	0.02 [0.01,0.04]	0.73 [0.72,0.74]
	**Combined**	0.56 [0.54,0.58]		0.34 [0.31,0.36]		0.73 [0.72,0.74]	
Women	Smoking	0.26 [0.25,0.27]	0.26 [0.25,0.27]	-	-	0.55 [0.54,0.56]	0.55 [0.54,0.56]
	Diet	0.14 [0.11,0.17]	0.36 [0.34,0.38]	0.12 [0.10,0.15]	0.12 [0.10,0.15]	0.16 [0.13,0.19]	0.63 [0.61,0.64]
	High blood pressure	0.12 [0.10,0.14]	0.44 [0.42,0.46]	0.13 [0.11,0.15]	0.24 [0.21,0.27]	0.11 [0.09,0.13]	0.67 [0.65,0.68]
	Physical inactivity	0.06 [0.03,0.09]	0.47 [0.44,0.50]	0.06 [0.03,0.09]	0.29 [0.25,0.32]	0.06 [0.03,0.09]	0.69 [0.67,0.70]
	Alcohol intake	0.02 [0.01,0.03]	0.48 [0.45,0.51]	0.01 [0.01,0.02]	0.29 [0.26,0.33]	0.03 [0.02,0.04]	0.70 [0.68,0.71]
	Overweight and obesity (BMI)	0.04 [0.02,0.06]	0.50 [0.47,0.53]	0.05 [0.03,0.06]	0.33 [0.29,0.36]	0.03 [0.02,0.04]	0.71 [0.69,0.72]
	**Combined**	0.50 [0.47,0.53]		0.33 [0.29,0.36]		0.71 [0.69,0.72]	
Men	Smoking	0.38 [0.37,0.39]	0.38 [0.37,0.39]	-	-	0.58 [0.57,0.58]	0.58 [0.57,0.58]
	Diet	0.14 [0.10,0.17]	0.46 [0.44,0.49]	0.13 [0.09,0.17]	0.13 [0.09,0.17]	0.14 [0.10,0.18]	0.64 [0.62,0.66]
	High blood pressure	0.13 [0.09,0.16]	0.54 [0.51,0.56]	0.13 [0.10,0.16]	0.24 [0.20,0.28]	0.11 [0.08,0.15]	0.69 [0.67,0.71]
	Physical inactivity	0.08 [0.05,0.10]	0.57 [0.55,0.60]	0.08 [0.05,0.10]	0.30 [0.25,0.34]	0.08 [0.05,0.10]	0.71 [0.69,0.73]
	Alcohol intake	0.06 [0.05,0.08]	0.60 [0.57,0.63]	0.04 [0.03,0.05]	0.33 [0.28,0.37]	0.08 [0.06,0.10]	0.74 [0.72,0.76]
	Overweight and obesity (BMI)	0.03 [0.01,0.06]	0.61 [0.59,0.64]	0.03 [0.01,0.06]	0.35 [0.30,0.39]	0.03 [0.00,0.05]	0.75 [0.73,0.76]
	**Combined**	0.61 [0.59,0.64]		0.35 [0.30,0.39]		0.75 [0.73,0.76]	

[1] Attributable fractions were calculated based on the difference in expected cumulative risk given the observed covariate distributions in EPIC and the expected cumulative risk under the following scenarios. **Smoking:** A population of never smokers. **Diet:** A population of people in the *healthy* category. **Blood pressure**: A population of people with normal blood pressure. **High alcohol intake:** A population who drink at most 1-2 drinks per day. **Physical Activity:** A population of people in the *active* category. **Overweight and obesity:** A population of people with BMI no higher than 25. These attributable risks thus represent a best case, in that they are calculated based on a hypothetical reference population with risk factors removed entirely.
[2] Estimated using predictions from a model mutually adjusted for all listed covariates as well as age at baseline. Attributable fractions are based on modifying one covariate at a time, with the distribution of the remaining covariates left as observed in EPIC.
[3] The cumulative attributable fraction after the sequential addition of each covariate.
