# Supplementary material for: Modifiable causes of premature death in middle-age in Western Europe: results from the EPIC cohort study
Source: BMC Med. 2016 Jun 14;14:87. doi: 10.1186/s12916-016-0630-6 (PMC4907105; doi:10.1186/s12916-016-0630-6)
Supplement: Additional file 3: Methods 1. — Supplementary Methods for “Modifiable causes of death in middle-age in Western Europe: results from the EPIC cohort study”. (PDF 100 kb) [file 12916_2016_630_MOESM3_ESM.pdf]

S1 Methods. Supplementary Methods for “Modifiable causes of death in middle-age in Western Europe: results from the EPIC cohort study”

Definition of the WCRF diet score

The dietary score used was an adapted version of the WCRF/AICR score (4), including the intakes of energy dense foods/sugary drinks, plant foods (fruits/vegetables/dietary fibre), and animal foods (red and processed meat) only. These dietary components were graded according to the cut-points detailed in the table below. Based on this scoring, participant diets were graded from 0 to 3; from which sex-specific fourths were created: (1) unhealthy; (2) moderately unhealthy; (3) moderately healthy; (4) healthy.

WCRF/AICR recommendations for cancer prevention and operationalization of the WCRF/AICR score in the EPIC (4) study

Dietary components and WCRF personal recommendations	Exposure category definition	Scoring
**Foods and drinks that promote weight gain. Limit consumption of energy-dense foods; avoid sugary drinks.**		
3a) Consume energy-dense foods sparingly.*	ED: $\leq 125\text{kcal} \cdot 100\text{g}^{-1} \cdot \text{day}^{-1}$	0.5
	ED: $> 125\text{to} \leq 175\text{kcal} \cdot 100\text{g}^{-1} \cdot \text{day}^{-1}$	0.25
	ED: $> 175\text{kcal} \cdot 100\text{g}^{-1} \cdot \text{day}^{-1}$	0
3b) Avoid sugary drinks.	Sugary drink intake: 0 g/day	0.5
	Sugary drink intake: $\leq$250 g/day	0.25
	Sugary drink intake: $>$250 g/day	0
**Plant foods. Eat mostly foods of plant origin.**		
4a) Eat 5 portions/servings (400 g) of a variety of non-starchy vegetables and of fruit every day.	F&V intake: $\geq$400 g/d	0.5
	F&V intake: 200 to $<$400 g/day	0.25
	F&V intake: $<$200 g/day	0
4b) Eat relatively unprocessed cereals (grains) and/or pulses (legumes) with every meal.	Dietary fibre intake: 25 g/d	0.5
	Dietary fibre intake: 12.5 to $<$25 g/day	0.25
	Dietary fibre intake: $<$12.5 g/day	0
**Animal foods. Limit intake of red meat and avoid processed meat.**		
5a) People who eat red meat should consume $<$500 g/wk and very few, if any, processed meats.	Red and processed meat $<$500 g/wk and processed meat intake $<$3 g/d	1
	Red and processed meat $<$500 g/wk and processed meat intake 3 to $<$50 g/d	0.5
	Red and processed meat $\geq$500 g/wk or processed meat intake $\geq$50 g/d	0

*Energy density (ED) was calculated as energy (kcal) from all foods (solid foods and semi-solid or liquid foods such as soups) divided by the weights (g) of these foods. Drinks (including water, tea, coffee, juice, soft drinks, alcoholic drinks and milk) were not included in the calculation

Attributable fraction calculations: technical details

We wished to estimate the attributable fraction, or the proportion of incident deaths that would not have occurred under the hypothetical circumstance in which the covariates have different distributions to those that were observed. For example, the proportion of deaths that would be prevented if the population consisted of only never-smokers, rather than a mix of never, former, and current-smokers. Let $\mathbf{X}$ denote the matrix of covariates as observed, and $\mathbf{X}^*$ denote a matrix of the same covariates that has been *intervened upon* such that one or more of the covariates have a different (counterfactual) distribution to that observed. We define an attributable fraction function in terms of expectations of the model-based cumulative incidence function for death ($\hat{F}$):

$$AF(t;\mathbf{X},\hat{\theta}) = \frac{\mathbb{E}[\hat{F}(t;\mathbf{X},\hat{\theta})] - \mathbb{E}[\hat{F}(t;\mathbf{X}^*,\hat{\theta})]}{\mathbb{E}[\hat{F}(t;\mathbf{X},\hat{\theta})]}.$$

Where t is time-scale of the survival model (in this case, age), and $\hat{\theta}$ is the vector of parameter estimates for the survival model. In practice, $\mathbb{E}[\hat{F}(t;\mathbf{X})]$ is calculated by making predictions from the fitted model given the observed covariate values for each participant, and then taking the mean of these values. $\mathbb{E}[\hat{F}(t;\mathbf{X}^*)]$ is calculated similarly, but with predictions based on a counterfactual distribution for the covariates that reflects the removal of one or more risk factors. Confidence intervals for AF can be calculated based on the delta method estimate of the variance of $\log(-\log(AF))$.
