# Supplementary figures and images for: Modifiable causes of premature death in middle-age in Western Europe: results from the EPIC cohort study
Source: BMC Med. 2016 Jun 14;14:87. doi: 10.1186/s12916-016-0630-6 (PMC4907105; doi:10.1186/s12916-016-0630-6)

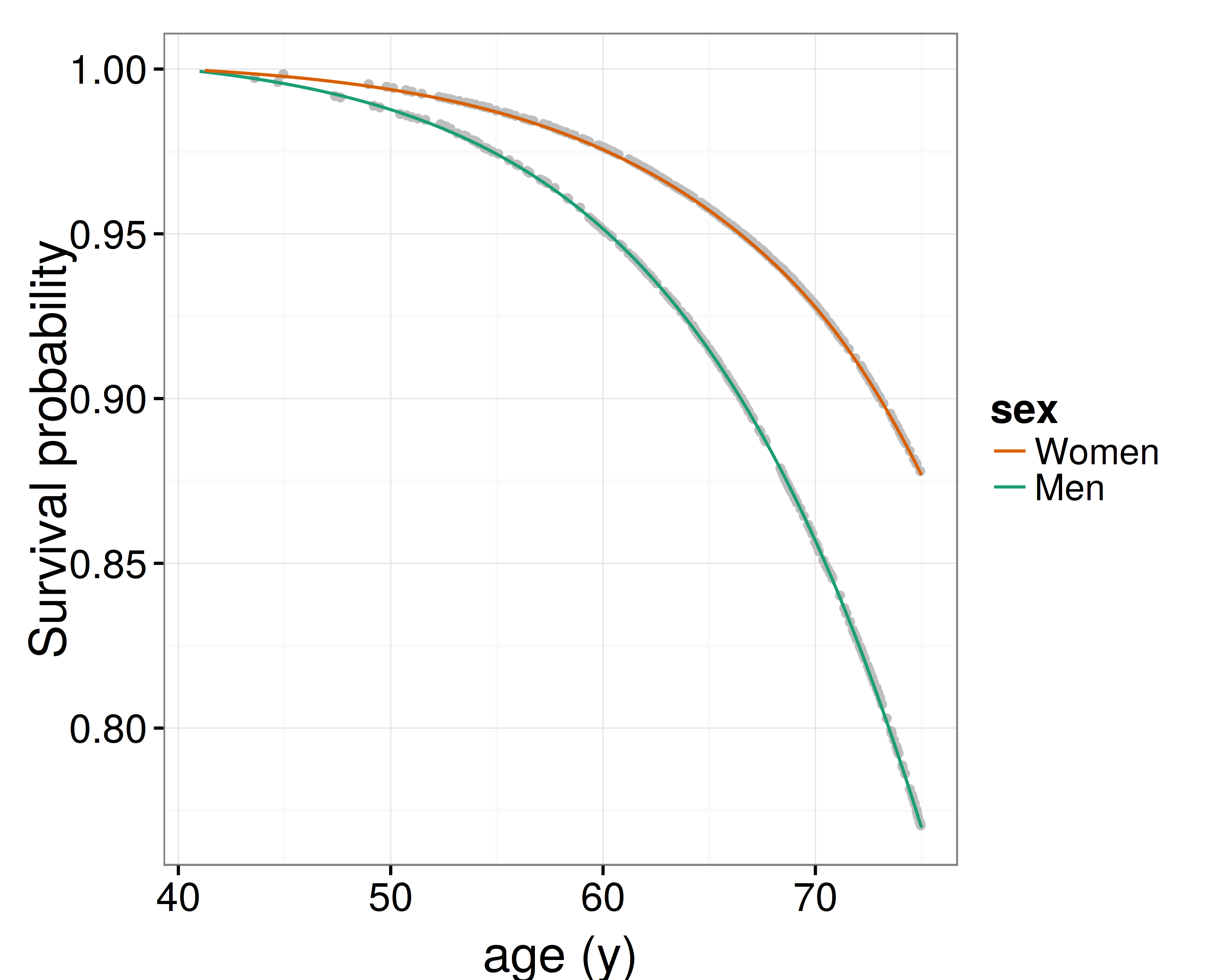

Supplement: Additional file 4: Figure S1. — Model-based survival functions for men and women in EPIC (coloured lines) and a random sample of Kaplan–Meier estimates of the survival function evaluated at failure times (grey dots). Survival to age 70 years in the EU in general was estimated to be 0.89 for women and 0.80 for men (using mortality rates from 2006–2010 obtained from http://ec.europa.eu/eurostat). (TIFF 402 kb) [file 12916_2016_630_MOESM4_ESM.tiff]

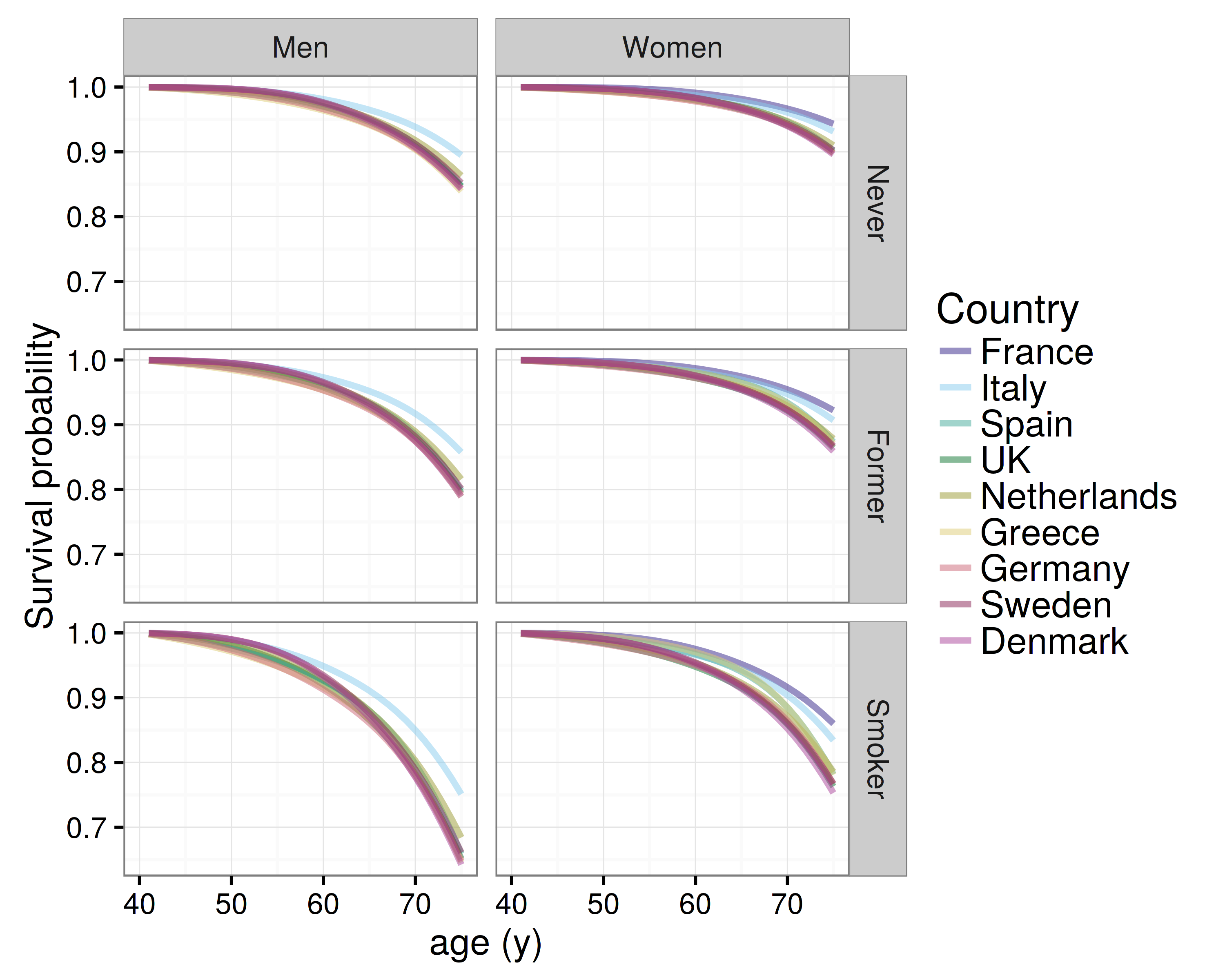

Supplement: Additional file 5: — Model -based survival functions for each country in EPIC, by sex and smoking status. (TIFF 824 kb) [file 12916_2016_630_MOESM5_ESM.tiff]
