# Supplementary material for: Modifiable causes of premature death in middle-age in Western Europe: results from the EPIC cohort study
Source: BMC Med. 2016 Jun 14;14:87. doi: 10.1186/s12916-016-0630-6 (PMC4907105; doi:10.1186/s12916-016-0630-6)
Supplement: Additional file 6: Table S1. — Number of participants in the EPIC cohort: overall, for premature deaths (prior to age 70 years), by country of recruitment and sex. (PDF 32 kb) [file 12916_2016_630_MOESM6_ESM.pdf]

S1 Table. Number of participants in the EPIC cohort: overall, for premature deaths (prior to age 70 years), by country of recruitment and sex.

	Total		Premature deaths		Sex			
					Male		Female	
Country	n	percent	n	percent	n	percent	n	percent
Denmark	56682	21	4058	34	26988	29	29694	17
Italy	39357	15	1152	10	11909	13	27448	16
UK	36700	14	1470	12	13592	15	23108	13
Germany	30770	12	1252	10	14305	15	16465	10
Sweden	27943	11	1599	13	10984	12	16959	10
Netherlands	26827	10	1143	10	4729	5	22098	13
Greece	20543	8	582	5	8241	9	12302	7
France	20454	8	457	4	0	0	20454	12
Spain	5630	2	217	2	2039	2	3591	2
Norway	0	0	0	0	0	0	0	0

Blood pressure was not collected in Norway, so no participants from Norway contributed to this analysis.
