# Supplementary material for: Modifiable causes of premature death in middle-age in Western Europe: results from the EPIC cohort study
Source: BMC Med. 2016 Jun 14;14:87. doi: 10.1186/s12916-016-0630-6 (PMC4907105; doi:10.1186/s12916-016-0630-6)
Supplement: Additional file 7: Table S2. — Attributable fractions of deaths prior to age 70 years given the distribution of covariates in the EPIC cohort, using body mass index to assess overweight and obesity. (PDF 49 kb) [file 12916_2016_630_MOESM7_ESM.pdf]

S2 Table. Distribution of covariates by country of recruitment.

		Country																	
		Denmark		Italy		UK		Germany		Sweden		Netherlands		Greece		France		Spain	
		n	%	n	%	n	%	n	%	n	%	n	%	n	%	n	%	n	%
Sex	Male	26988	48	11909	30	13592	37	14305	46	10984	39	4729	18	8241	40	0	0	2039	36
	Female	29694	52	27448	70	23108	63	16465	54	16959	61	22098	82	12302	60	20454	100	3591	64
Age at baseline (years)	[40,50)	0	0	16119	41	10597	29	12455	40	5333	19	6167	23	6182	30	8031	39	2345	42
	[50,60)	41451	73	17349	44	12279	33	11763	38	10859	39	14126	53	5648	27	8889	43	2193	39
	[60,70]	15231	27	5697	14	10294	28	6551	21	9098	33	6530	24	6619	32	3528	17	1092	19
Smoking	Never smoker	19838	35	18303	47	18082	49	14583	47	10609	38	10091	38	12240	60	14437	71	3560	63
	Former smoker	17292	31	10482	27	13790	38	10158	33	9449	34	9339	35	3599	18	4376	21	906	16
	Current smoker	19552	34	10572	27	4828	13	6029	20	7885	28	7397	28	4704	23	1641	8	1164	21
Blood pressure	normal	8063	14	8553	22	8504	23	6234	20	2510	9	7580	28	3939	19	8003	39	1549	28
	Pre-hypert.	19981	35	16782	43	14705	40	12669	41	9171	33	11041	41	7450	36	8184	40	2280	40
	Hypert. 1	19080	34	10074	26	9416	26	8221	27	9647	35	5477	20	6010	29	3271	16	1233	22
	Hypert. 2	9558	17	3948	10	4075	11	3646	12	6615	24	2729	10	3144	15	996	5	568	10
BMI (kg/m^2)	<20	2029	4	1322	3	1619	4	862	3	1581	6	1207	4	234	1	3113	15	34	1
	20-21.9	5805	10	4146	11	4674	13	2674	9	3690	13	3317	12	743	4	5187	25	140	2
	22-24.9 (reference)	17086	30	11733	30	11950	33	8345	27	8931	32	8655	32	3268	16	6857	34	855	15
	25-29.9 (overweight)	23531	42	16166	41	13930	38	13193	43	10435	37	10218	38	9000	44	4179	20	2727	48
	30-34.9 (obese)	6529	12	4721	12	3544	10	4443	14	2691	10	2710	10	5367	26	892	4	1373	24
	35+ (very obese)	1702	3	1269	3	983	3	1253	4	615	2	720	3	1931	9	226	1	501	9
Waist-to-hip ratio (sex-specific fifths)	1	9965	18	8639	22	11763	32	6134	20	5042	18	6297	23	2788	14	6312	31	396	7
	2	11073	20	7650	19	8431	23	5809	19	6412	23	5082	19	3013	15	4369	21	849	15
	3	12412	22	9021	23	7613	21	6674	22	7542	27	6321	24	4003	19	5135	25	1245	22
	4	10047	18	7073	18	4787	13	5567	18	4962	18	4530	17	3969	19	2663	13	1468	26
	5	13185	23	6974	18	4106	11	6586	21	3985	14	4597	17	6770	33	1975	10	1672	30
Alcohol intake (drinks/day)	0	1318	2	6752	17	4462	12	1186	4	4676	17	4148	15	5596	27	2516	12	2172	39
	(0,.5]	11117	20	11997	30	14240	39	9934	32	6968	25	9427	35	7217	35	6708	33	1263	22
	(0.5,1]	9546	17	3068	8	7989	22	5727	19	5780	21	3409	13	2656	13	3518	17	465	8
	(1,2]	15191	27	6658	17	5908	16	5572	18	6427	23	4174	16	2405	12	3942	19	627	11
	(2,6]	15858	28	9839	25	3860	11	7175	23	3924	14	5235	20	2159	11	3507	17	911	16
	>6 (women), (6,10] (men)	3160	6	956	2	224	1	1008	3	152	1	383	1	378	2	263	1	159	3
	>10 (men)	492	1	87	0	17	0	168	1	16	0	51	0	132	1	0	0	33	1
Diet	unhealthy	7049	12	852	2	3225	9	2260	7	5819	21	2414	9	72	0	570	3	85	2
	moderately unhealthy	28959	51	11994	30	15186	41	18189	59	14616	52	13434	50	2278	11	7094	35	1215	22
	moderately healthy	14548	26	16018	41	10100	28	7637	25	5342	19	7406	28	6004	29	7770	38	2285	41
	healthy	6126	11	10493	27	8189	22	2684	9	2166	8	3573	13	12189	59	5020	25	2045	36
Physical activity	inactive	6294	11	12166	31	12108	33	5978	19	6449	23	2421	9	10269	50	3575	17	2511	45
	moderately inactive	17299	31	15058	38	12078	33	11448	37	10691	38	7227	27	4908	24	8409	41	1789	32
	moderately active	13767	24	6724	17	7428	20	7739	25	6407	23	7012	26	3974	19	6655	33	842	15
	active	19322	34	5409	14	5086	14	5605	18	4396	16	10167	38	1392	7	1815	9	488	9
