# Supplementary material for: Modifiable causes of premature death in middle-age in Western Europe: results from the EPIC cohort study
Source: BMC Med. 2016 Jun 14;14:87. doi: 10.1186/s12916-016-0630-6 (PMC4907105; doi:10.1186/s12916-016-0630-6)
Supplement: Additional file 8: Table S5. — Hazard ratios and confidence intervals for death prior to age 70 years in the EPIC cohort. (PDF 76 kb) [file 12916_2016_630_MOESM8_ESM.pdf]

S5 Table. Population attributable fractions of deaths prior to age 75 given the distribution of covariates in the EPIC cohort, using waist-to-hip ratio to assess for obesity.

		All participants		Never-smokers		Current-smokers	
	Covariate[1]	Overall[2]	Cumulative[3]	Overall[2]	Cumulative[3]	Overall[2]	Cumulative[3]
Women and Men	Smoking	0.30 [0.30,0.31]	0.30 [0.30,0.31]	-	-	0.55 [0.54,0.56]	0.55 [0.54,0.56]
	Diet	0.13 [0.11,0.16]	0.40 [0.38,0.41]	0.12 [0.10,0.14]	0.12 [0.10,0.14]	0.14 [0.12,0.17]	0.62 [0.61,0.63]
	Overweight and obesity (WHR)	0.09 [0.08,0.11]	0.46 [0.44,0.47]	0.09 [0.08,0.11]	0.20 [0.18,0.23]	0.09 [0.07,0.11]	0.66 [0.65,0.67]
	High blood pressure	0.09 [0.07,0.10]	0.51 [0.49,0.52]	0.09 [0.07,0.11]	0.27 [0.25,0.30]	0.08 [0.06,0.09]	0.69 [0.68,0.70]
	Physical inactivity	0.07 [0.05,0.09]	0.54 [0.52,0.56]	0.08 [0.06,0.10]	0.33 [0.30,0.36]	0.06 [0.04,0.08]	0.71 [0.70,0.72]
	Alcohol intake	0.03 [0.03,0.04]	0.56 [0.54,0.58]	0.02 [0.01,0.02]	0.34 [0.31,0.37]	0.05 [0.04,0.05]	0.73 [0.71,0.74]
	**Combined**	0.56 [0.54,0.58]		0.34 [0.31,0.37]		0.73 [0.71,0.74]	
Women	Smoking	0.26 [0.25,0.26]	0.26 [0.25,0.26]	-	-	0.55 [0.54,0.56]	0.55 [0.54,0.56]
	Diet	0.13 [0.10,0.16]	0.35 [0.33,0.38]	0.12 [0.09,0.15]	0.12 [0.09,0.15]	0.15 [0.12,0.18]	0.62 [0.60,0.64]
	Overweight and obesity (WHR)	0.07 [0.04,0.09]	0.40 [0.37,0.42]	0.07 [0.04,0.09]	0.18 [0.15,0.21]	0.07 [0.04,0.09]	0.65 [0.63,0.67]
	High blood pressure	0.09 [0.07,0.11]	0.46 [0.43,0.48]	0.10 [0.08,0.12]	0.26 [0.23,0.30]	0.08 [0.06,0.10]	0.68 [0.66,0.70]
	Physical inactivity	0.06 [0.03,0.09]	0.49 [0.46,0.52]	0.06 [0.03,0.10]	0.31 [0.27,0.35]	0.05 [0.02,0.08]	0.70 [0.68,0.72]
	Alcohol intake	0.02 [0.01,0.02]	0.50 [0.47,0.52]	0.01 [0.01,0.02]	0.32 [0.28,0.35]	0.02 [0.01,0.03]	0.71 [0.69,0.72]
	**Combined**	0.50 [0.47,0.52]		0.32 [0.28,0.35]		0.71 [0.69,0.72]	
Men	Smoking	0.35 [0.33,0.36]	0.35 [0.33,0.36]	-	-	0.54 [0.53,0.56]	0.54 [0.53,0.56]
	Diet	0.13 [0.09,0.16]	0.43 [0.41,0.46]	0.13 [0.09,0.16]	0.13 [0.09,0.16]	0.13 [0.09,0.17]	0.61 [0.59,0.63]
	Overweight and obesity (WHR)	0.12 [0.10,0.15]	0.51 [0.48,0.54]	0.11 [0.09,0.14]	0.22 [0.18,0.26]	0.12 [0.10,0.14]	0.67 [0.64,0.69]
	High blood pressure	0.08 [0.04,0.11]	0.55 [0.52,0.58]	0.08 [0.04,0.11]	0.29 [0.24,0.33]	0.06 [0.03,0.10]	0.69 [0.67,0.71]
	Physical inactivity	0.07 [0.04,0.09]	0.59 [0.55,0.61]	0.07 [0.04,0.09]	0.34 [0.29,0.38]	0.06 [0.04,0.09]	0.72 [0.69,0.74]
	Alcohol intake	0.05 [0.04,0.07]	0.61 [0.58,0.64]	0.03 [0.02,0.05]	0.36 [0.31,0.40]	0.07 [0.05,0.08]	0.74 [0.72,0.76]
	**Combined**	0.61 [0.58,0.64]		0.36 [0.31,0.40]		0.74 [0.72,0.76]	

[1] Attributable fractions were calculated based on the difference in expected cumulative risk given the observed covariate distributions in EPIC and the expected cumulative risk under the following scenarios. **Smoking:** A population of never smokers. **Diet:** A population of people in the *healthy* category. **Blood pressure**: A population of people with normal blood pressure. **High alcohol intake:** A population who drink at most 1-2 drinks per day. **Physical Activity:** A population of people in the *active* category. **Overweight and obesity:** A population of people with WHR below the lowest sex-specific quintile. These attributable risks thus represent a best case, in that they are calculated based on a hypothetical reference population with risk factors removed entirely.
[2] Estimated using predictions from a model mutually adjusted for all listed covariates as well as age at baseline. Attributable fractions are based on modifying one covariate at a time, with the distribution of the remaining covariates left as observed in EPIC.
[3] The cumulative attributable fraction after the sequential addition of each covariate.
