# Supplementary material for: Modifiable causes of premature death in middle-age in Western Europe: results from the EPIC cohort study
Source: BMC Med. 2016 Jun 14;14:87. doi: 10.1186/s12916-016-0630-6 (PMC4907105; doi:10.1186/s12916-016-0630-6)
Supplement: Additional file 10: Table S7. — Hazard ratios and confidence intervals for death prior to age 70 years in the EPIC cohort. (PDF 42 kb) [file 12916_2016_630_MOESM10_ESM.pdf]

S7 Table. Hazard ratios and confidence intervals for death prior to age 70 years in the EPIC cohort.

		Overall		Women		Men	
		HR	[95% CI]	HR	[95% CI]	HR	[95% CI]
Total/HDL cholesterol (sex-specific fourths)	< 3.79 (men), < 3.09 (women)	1.00		1.00		1.00	
	[3.79,4.64) (men), [3.09,3.76) (women)	1.25	[0.91,1.69]	1.49	[0.96,2.32]	1.05	[0.68,1.62]
	[4.64,5.73) (men), [3.76,4.69) (women)	1.44	[1.06,1.94]	1.32	[0.84,2.08]	1.57	[1.04,2.35]
	5.73 + (men), 4.69 + (women)	1.65	[1.23,2.23]	1.74	[1.11,2.73]	1.63	[1.09,2.45]
Glycated haemoglobin (sex-specific fourths)	< 5.26	1.00		1.00		1.00	
	[5.26,5.54) (men), [5.26,5.44) (women)	0.88	[0.67,1.15]	1.10	[0.75,1.62]	0.74	[0.51,1.07]
	[5.54,5.72) (men), [5.44,5.72) (women)	0.91	[0.69,1.20]	0.89	[0.61,1.30]	0.95	[0.63,1.42]
	5.72 +	1.06	[0.82,1.38]	1.01	[0.69,1.50]	1.11	[0.78,1.57]

Estimates from flexible parametric survival models with attained age as the time-scale. Separate models were fitted cholesterol and glycated haemoglobin. All models included age at baseline attendance, country of recruitment, smoking status, physical activity, diet, alcohol intake, and BMI. Overall estimates are also adjusted for sex.
